# Multidisciplinary management of surgery and postoperative recurrence in stage IV gallbladder cancer following conversion therapy: a case report

**DOI:** 10.3389/fonc.2025.1749491

**Published:** 2026-01-07

**Authors:** Junfeng Cheng, Xuemin Li, Chao Ren, Shian Yu, Hongwei Huang, Shishi Zhou, Jianji Dai

**Affiliations:** 1 Department of Hepatopancreatobiliary Surgery, Jinhua Municipal Central Hospital, Jinhua, China; 2 Department of Radiology, Jinhua Municipal Central Hospital, Jinhua, China; 3 Department of Medical Oncology, Jinhua Municipal Central Hospital, Jinhua, China; 4 Department of Intervention, Jinhua Municipal Central Hospital, JJinhua, China

**Keywords:** case report, conversion therapy, gallbladder cancer, multidisciplinary management, radiofrequency ablation

## Abstract

A 61-year-old female patient presented with a 10-day history of right upper abdominal pain and was subsequently diagnosed with stage IV gallbladder cancer based on preoperative imaging and pathological evaluation. Following multidisciplinary discussion, a triple-agent combination therapy was initiated. Although the patient developed severe grade II cutaneous toxicity during treatment, a significant clinical response was achieved, and symptoms were effectively relieved with supportive management. After four months of conversion therapy, the patient underwent successful radical surgical resection. Five months postoperatively, isolated hepatic metastases were identified, and a multidisciplinary team subsequently recommended radiofrequency ablation. The patient is currently receiving maintenance therapy with immune checkpoint inhibitors. At the most recent follow-up, the patient remains disease-free, with an overall survival of 18 months.

## Introduction

Gallbladder cancer (GBC) is the most prevalent malignancy of the biliary tract and is characterized by an insidious onset, aggressive biological behavior, and poor prognosis (1, 2). Owing to the absence of early symptoms, the disease is often diagnosed at an advanced stage, at which point surgical resection is no longer feasible, leading to significantly short survival. In recent years, the emergence of conversion therapy (3–6) and advances in comprehensive systemic treatment have promoted the combined use of small-molecule targeted agents and immune checkpoint inhibitors.

This therapeutic strategy has shown encouraging outcomes in cancer treatment, with accumulating evidence demonstrating its synergistic effects and clinical efficacy (7, 8). Some patients with advanced malignancies have achieved substantial survival benefits, with median overall survival reaching 38.3 months with targeted therapy and up to 15.7 months with immunotherapy as first-line treatment, creating opportunities for subsequent surgical intervention and prolonged survival (9, 10). However, conversion therapy for gallbladder cancer has thus far been reported mainly in isolated case reports, with a lack of large-scale clinical studies (11–13). Here, we present a recent case of stage IV GBC treated at our institution, in which the patient achieved 18 months of survival following surgery and postoperative recurrence, surpassing the expected survival for this disease stage under multidisciplinary evaluation and management.

## Case presentation

**Figure 1:**
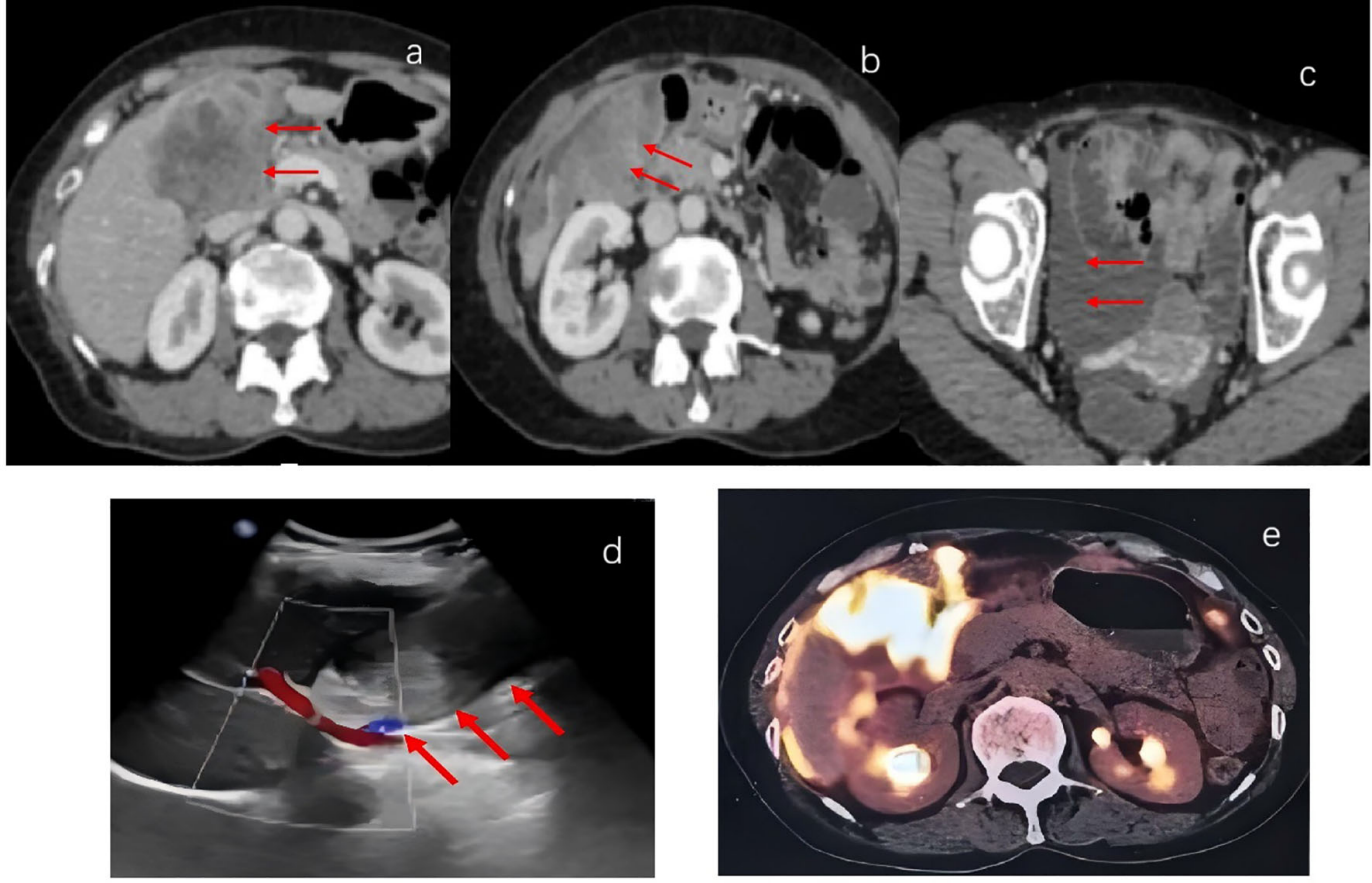
Baseline imaging before treatment: **(a-c)** Contrast-enhanced abdominal computed tomography (CT) scans images: A large mass was observed in the gallbladder fossa(red arrow), with invasion of the adjacent liver parenchyma and hepatocolic region; omentum thickening and multiple enlarged abdominal cavity lymph nodes, indicative of metastasis accompanied by ascites(red arrow);**(d)** Ultrasound images: A mass measuring 9.8 × 4.4 cm was observed in the gallbladder fossa, with an internal hypoechoic mass of irregular shape(red arrow); the gallbladder was not visible.**(e)** Positron emission tomography - computed tomography (PET-CT) images: the gallbladder tumor showed a maximum SUV uptake of 13.9, accompanied by peritoneal metastasis.

**Figure 2:**
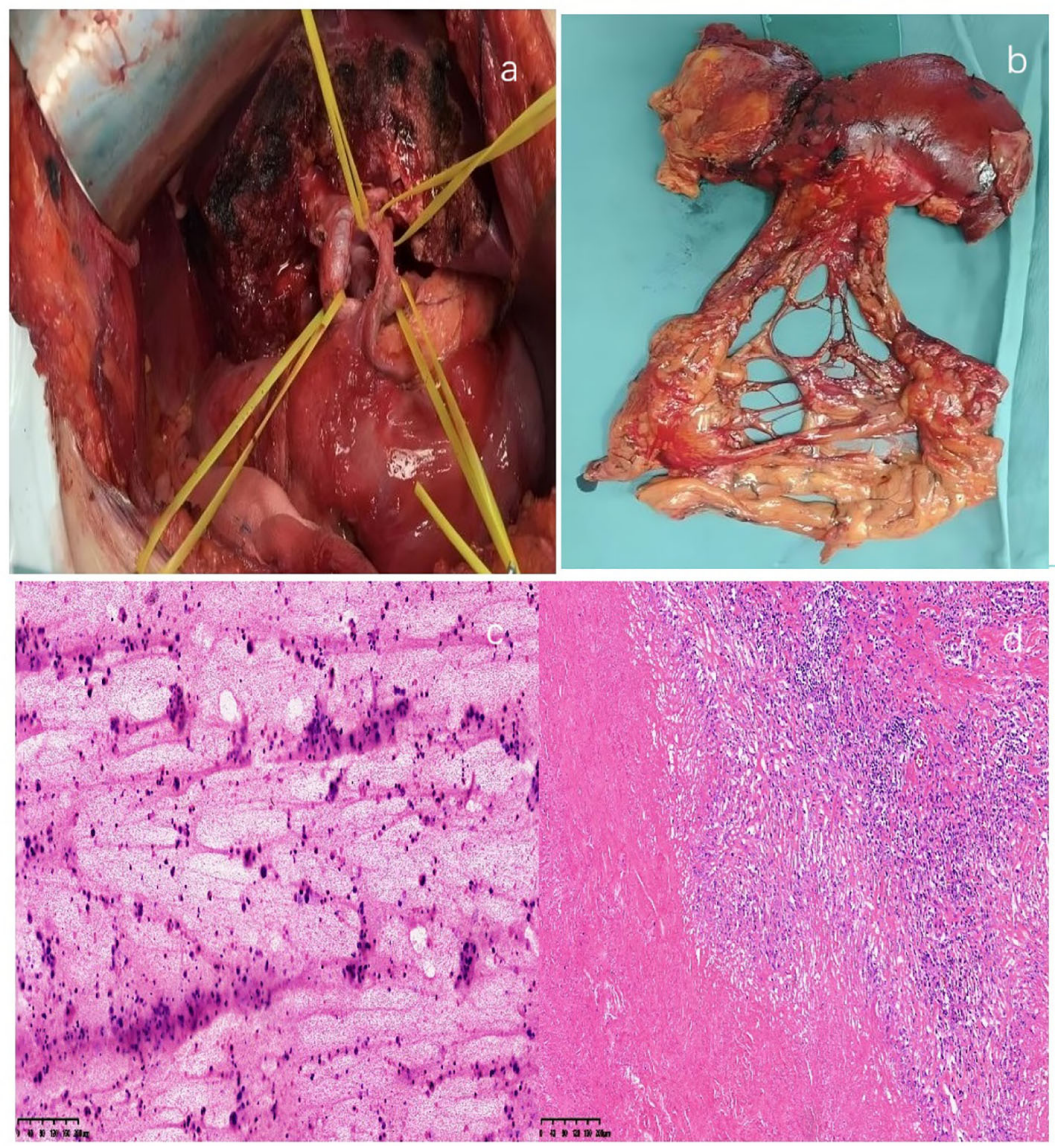
Surgery, specimen and pathology: **(a)** Intraoperative situation; **(b)** Postoperative specimen; **(c)** Initial diagnosis of adenocarcinoma in the gallbladder mass via puncture cytology before treatment, H&E staining ×100; **(d)** Post-treatment observation of extensive necrosis in gallbladder tissue, with no residual tumor cells (H&E staining ×100).

A 61-year-old woman presented in October 2023 with a 10-day history of right upper quadrant abdominal pain. Physical examination revealed no scleral icterus and identified a firm, tender, and mobile mass approximately the size of an adult fist in the right upper abdomen. Serum CA125 was significantly elevated at 244.35 U/mL, while carcinoembryonic antigen (CEA) and CA19–9 levels were within normal limits. B-ultrasonography demonstrated a 9.8 × 4.4 cm hypoechoic mass with peritoneal infiltration in the gallbladder fossa, with the gallbladder not clearly visualized (**Figure 1d**). Contrast-enhanced abdominal computed tomography (CT) scans revealed a large mass originating from the gallbladder with invasion of adjacent hepatic and colonic tissues, consistent with gallbladder carcinoma (**Figure 1a–c**). Multiple enlarged peritoneal lymph nodes, peritoneal and reticular metastases, and ascites were also noted. Positron emission tomography-computed tomography (PET-CT) further suggested gallbladder cancer with possible hepatic metastasis and colonic invasion, as well as suspected peritoneal, mesenteric, and retroperitoneal metastases (**Figure 1e**). Cytological analysis of a fine-needle aspiration biopsy from the gallbladder mass confirmed poorly differentiated adenocarcinoma (**Figure 2c**). The tumor was staged as cT4N1M1, corresponding to stage IV gallbladder carcinoma.

**Figure 3:**
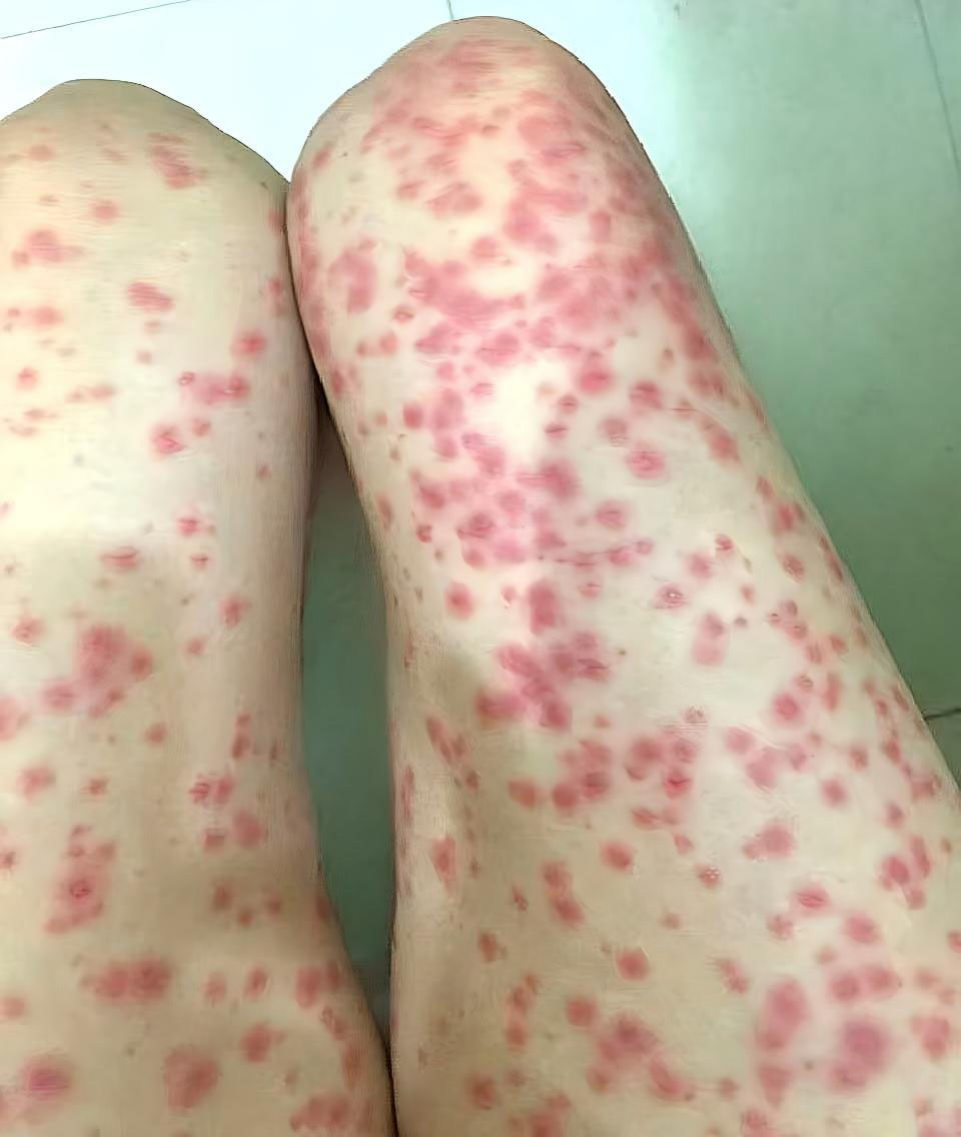
Skin adverse reactions in both lower limbs (multiple lesions throughout the body, including the face, abdomen, both lower limbs, anterior chest wall, and nape of the neck region).

Following an initial multidisciplinary evaluation, combination immunotherapy was initiated using a triple-drug regimen consisting of toripalimab (240 mg intravenously on day 1), lenvatinib (8 mg orally once daily), and the GEMOX chemotherapy protocol (gemcitabine 1.4 g intravenously on days 1 and 8, and oxaliplatin 110 mg intravenously on day 1, in a 21-day cycle). The patient received two cycles (C1–C2) of this regimen between 2 November 2023 and 10 January 2024. During treatment, she developed a severe generalized grade II erythematous rash with pruritus (**Figure 3**). After two weeks of multidisciplinary dermatologic consultation and anti-allergic therapy with intravenous methylprednisolone and oral loratadine, the symptoms gradually improved. However, owing to poor tolerance of the rash and pruritus, immune checkpoint inhibitor therapy was discontinued during cycles 3 and 4 (C3–C4) from February to April 2024, and treatment was continued with lenvatinib combined with the GEMOX regimen at the original doses. No further allergic reactions or rashes occurred thereafter, suggesting that the skin toxicity was associated with the immune checkpoint inhibitor.

**Figure 4:**
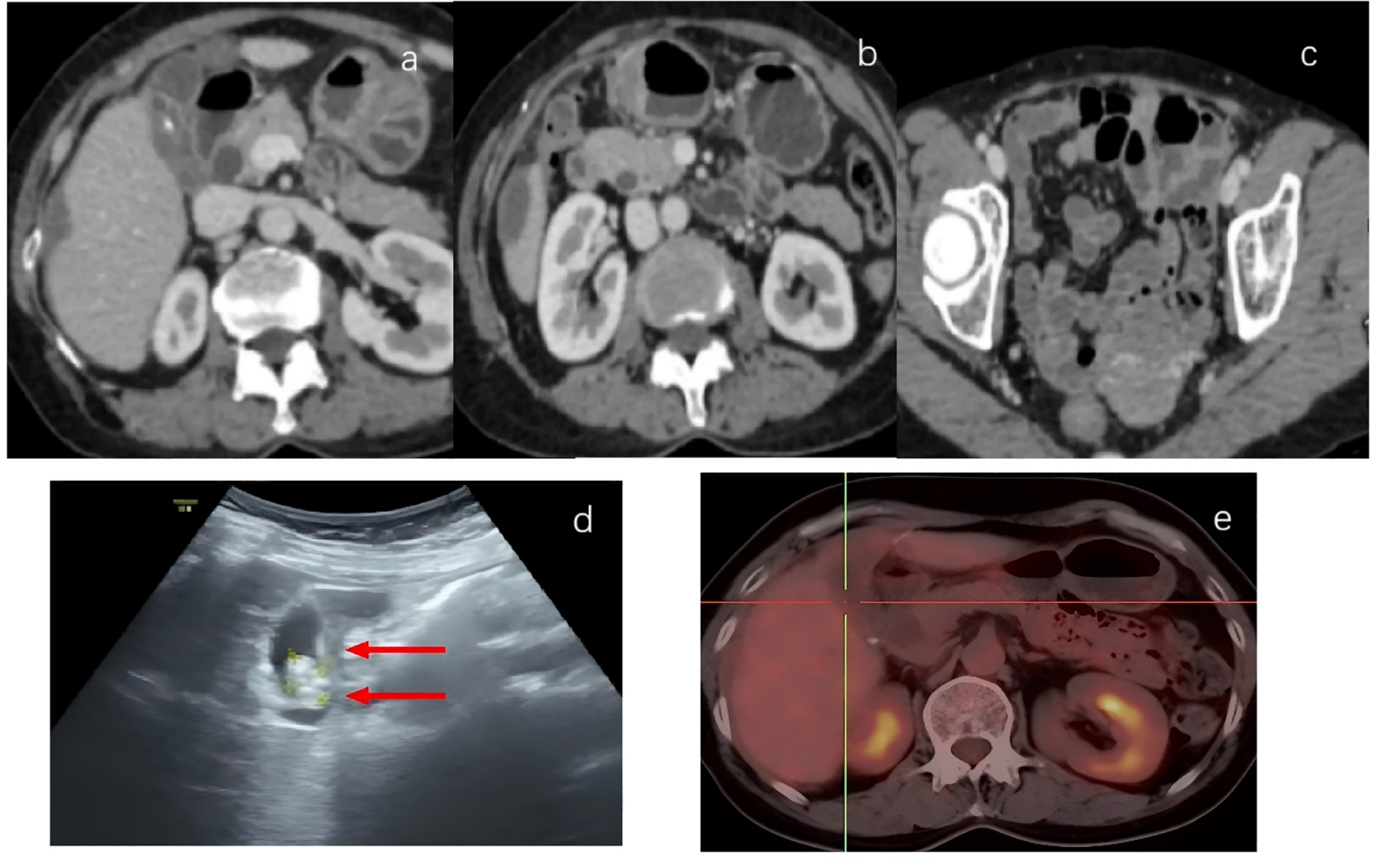
Post-conversion imaging: **(a–c)** Contrast-enhanced abdominal CT images: After conversion therapy figures **(a–c)** show post-treatment changes of gallbladder cancer, with mild gallbladder wall thickening and ascites resolution. **(d)** Ultrasound images: The gallbladder size was normal, the wall was thickened (red arrow), and two polypoid changes were visible in the cavity. **(e)** PET-CT images: the gallbladder showed hypermetabolism with a maximum SUV uptake of 2.9, which is within the normal range of gallbladder function.

Follow-up evaluation in April 2024, including ultrasonography and contrast-enhanced abdominal CT, demonstrated significant regression of the lesion with complete resolution of ascites (**Figures 4a–d**), and CA125 levels had normalized. PET-CT showed only focal increased FDG uptake in the gallbladder wall, indicating substantial improvement compared with baseline, while the peritoneal and omental lesions had significantly regressed. A low-density lesion beneath the right hepatic capsule with mild FDG uptake was considered metastatic (**Figure 4e**). After a second multidisciplinary discussion, laparoscopic exploration and extended cholecystectomy were performed on 23 April 2024 (**Figures 2a, b**). Postoperative pathology revealed post-treatment changes consistent with gallbladder cancer, a tumor regression grade of 0, no residual viable tumor cells, and no lymph node metastases (**Figure 2d**). The patient recovered uneventfully and was discharged without complications. From 10 May to 5 September 2024, she received three additional cycles of combined targeted therapy and chemotherapy followed by sequential immunotherapy, without recurrence of severe skin toxicity.

Five months after surgery, on 25 September 2024, liver MRI revealed a solitary recurrent metastatic lesion (**Supplementary Figures S1a, b**). Following a third multidisciplinary consultation, percutaneous local ablation of the hepatic metastasis was performed under local anesthesia on September 17, 2024, and the original triple-drug regimen was resumed. The patient subsequently underwent regular outpatient follow-up. Contrast-enhanced liver MRI in April 2025 demonstrated no evidence of tumor recurrence (**Supplementary Figures S1c, d**), and CA125 levels remained within the normal range. Owing to financial considerations, maintenance therapy was adjusted to immune checkpoint inhibitor monotherapy with toripalimab (240 mg intravenously on day 1 of a 21-day cycle). Currently, the patient has achieved 15 months of recurrence-free survival.

## Discussion

GBC is a highly aggressive malignancy of the digestive system, accounting for approximately one-third of biliary tract cancers, and is associated with an extremely poor long-term prognosis, with 5-year survival rates below 5% (14, 15). Surgical resection remains the only potentially curative option; however, most patients are diagnosed at intermediate or advanced stages, when surgical intervention is no longer feasible (16). As a result, effective systemic therapy relies heavily on multidisciplinary consultation and comprehensive therapeutic strategies.

With the increasing application of conversion therapy incorporating targeted agents and immune checkpoint inhibitors in advanced hepatocellular carcinoma (17), the integration of systemic treatment with surgery has improved resection rates in patients with advanced GBC, improving prognosis and prolonging survival (9). In this case, the patient showed extensive abdominal and peritoneal metastases at initial diagnosis, rendering radical resection impossible.

Immunotherapy-based combination regimens have become a major research focus in the treatment of advanced hepatobiliary malignancies. Most current studies have adopted dual therapeutic strategies, particularly immunotherapy combined with chemotherapy. The TOPAZ-1 trial, a global, randomized, double-blind, placebo-controlled phase III study, was the first to evaluate durvalumab combined with gemcitabine and cisplatin as first-line therapy for advanced biliary tract cancer (BTC). The study involving in 171 patients with GBC showed that the durvalumab-GC combination achieved a higher objective response rate (26.7% vs. 18.7%) and longer median overall survival (12.8 vs. 11.5 months) than GC alone (18). Subgroup analysis of the KEYNOTE-966 phase III trial (19), which assessed pembrolizumab plus chemotherapy as first-line therapy for BTC, reported an objective response rate of 36% and a median progression-free survival of 10.2 months in a Chinese population. Similarly, a “three-drug, four-agent” regimen combining gemcitabine and oxaliplatin (GEMOX) with trilaciclib and lenvatinib, developed by Academician Fan Jia’s team at Fudan Zhongshan Hospital (20), further confirmed the effectiveness of conversion strategies in advanced malignancies, with objective response rates as high as 80% and disease control rates of 93.3% in patients with GBC receiving conversion therapy. Lenvatinib inhibits the VEGF/FGFR signaling pathways, suppressing tumor angiogenesis, modulating the tumor immune microenvironment, and increasing immune cell infiltration. Gemcitabine combined with oxaliplatin induces immunogenic tumor cell death, promoting tumor antigen release and dendritic cell activation, generating a synergistic sequential effect with immune checkpoint inhibitors (21). In this case, tumor regression exceeded 90% following conversion therapy, enabling extended radical cholecystectomy and achieving a pathological complete response. These findings further support the effectiveness of the triple-drug combination regimen and suggest its potential to overcome immune tolerance in advanced GBC.

However, treatment-related adverse events must be carefully monitored, particularly cutaneous reactions, which require differentiation among gemcitabine-associated rashes, targeted therapy–induced hand-foot syndrome, and immune-mediated dermatitis (22). Immune checkpoint inhibitor–related skin toxicity typically manifests as self-limiting mild-to-moderate reactions (grades I–II) (23), usually occurring within 2–16 weeks after treatment initiation. These rashes generally present as diffuse erythema or papular eruptions that may coalesce into patchy lesions, most commonly involving the trunk and extensor surfaces of the limbs, and less frequently the face and neck. In comparison, gemcitabine-induced skin reactions display a characteristic temporal pattern, usually appearing 6.3 ± 1.3 days after initial administration, and primarily affect the anterolateral chest and abdominal wall. In rare cases, such reactions may progress to pseudocellulitis or skin necrosis (24, 25). In this case, the patient developed an extensive erythematous rash with intense pruritus on the first day of treatment, involving the limbs, trunk, abdominal wall, and face, making it initially difficult to distinguish immune-mediated dermatitis from gemcitabine hypersensitivity. Following repeated dermatologic consultations, combined intravenous methylprednisolone and oral loratadine effectively resolved the rash within two weeks. Nevertheless, the psychological burden of this adverse reaction led the patient to refuse toripalimab during the third and fourth cycles, after which similar rashes did not recur. These findings underscore the challenges of attributing treatment-related skin toxicity. At the same time, the favorable response to glucocorticoids and antihistamines highlights their effectiveness in managing rashes of diverse etiologies, although cautious use is warranted due to potential adverse effects.

Previous studies have shown that even after radical resection, most patients with intermediate- to advanced-stage GBC develop early recurrence or metastasis (14). In this case, oligometastatic liver recurrence occurred five months after R0 resection. Oligometastasis represents an intermediate biological state between localized disease and widespread metastasis. Recurrence may reflect the emergence of treatment-resistant tumor clones, whereas precise local interventions, such as radiofrequency ablation, can effectively eradicate resistant lesions and improve prognosis. Koshiro Morino et al. reported that patients with recurrent biliary tract tumors receiving local treatments, including radiofrequency ablation or radiotherapy, achieved significantly longer median survival than those receiving conventional therapy (15.2 vs. 8.7 months, P < 0.01), indicating that patients in the oligometastatic subgroup may derive substantial benefit from localized interventions (26). Accordingly, after a third multidisciplinary consultation, the patient underwent CT-guided radiofrequency ablation for hepatic metastasis. Due to financial constraints, maintenance therapy was continued with an immune checkpoint inhibitor monotherapy. To date, the patient has achieved a total survival of more than 15 months without evidence of tumor recurrence.

In summary, integrating targeted therapy with sequential surgical intervention can confer meaningful survival benefits in selected patients with advanced GBC. This case illustrates a stratified, multidisciplinary treatment strategy consisting of “systemic therapy for tumor downstaging, radical surgery, local ablation for oligometastatic recurrence, and maintenance immune checkpoint inhibition,” providing a personalized therapeutic pathway for advanced GBC. Future studies should focus on translational and clinical research to evaluate the generalizability of the triple-drug quadruple-agent regimen and identify reliable biomarkers to select patients most likely to benefit from targeted therapy.

## Data Availability

The original contributions presented in the study are included in the article/**Supplementary Material**. Further inquiries can be directed to the corresponding author.

## Supplementary material

Supplementary Figure 1 The magnetic resonance imaging (MRI) images: **(a-b)** After surgery, it showed a single metastatic lesion in the liver (red arrow); **(c-d)** After percutaneous local ablation of the hepatic metastasis and the original regimen of triple-drug therapy, it showed that the liver metastatic lesion had disappeared.
