# Supplementary figures and images for: Multidisciplinary management of surgery and postoperative recurrence in stage IV gallbladder cancer following conversion therapy: a case report
Source: Front Oncol. 2026 Jan 7;15:1749491. doi: 10.3389/fonc.2025.1749491 (PMC12819257; doi:10.3389/fonc.2025.1749491)

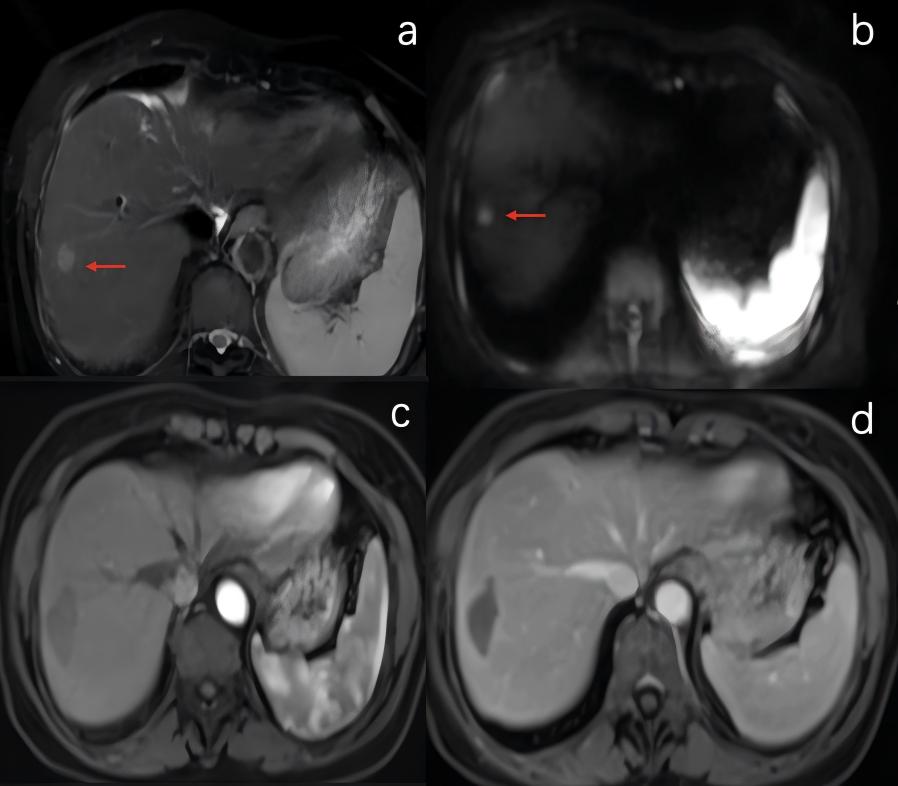

Supplement: Supplementary Figure 1 — The magnetic resonance imaging (MRI) images: (a-b) After surgery, it showed a single metastatic lesion in the liver (red arrow); (c-d) After percutaneous local ablation of the hepatic metastasis and the original regimen of triple-drug therapy, it showed that the liver metastatic lesion had disappeared. [file Image1.jpeg]
